# Impact of Metastatic Lymph Node Ratio on Survival and Prognosis in Rectal Carcinoma: A Retrospective Cohort Study

**DOI:** 10.7759/cureus.68734

**Published:** 2024-09-05

**Authors:** Bahadır Kartal, Mehmet Berksun Tutan

**Affiliations:** 1 General Surgery, Hitit University Erol Olçok Training and Research Hospital, Çorum, TUR; 2 General Surgery, Alaca State Hospital, Çorum, TUR

**Keywords:** localized rectal carcinoma, lymphovascular invasion, metastatic lymph node ratio, prognostic markers, survival data analysis

## Abstract

Objective

This study aimed to evaluate the impact of the metastatic lymph node ratio (mtLNR) on survival outcomes and prognosis in patients with rectal carcinoma, in comparison with other clinicopathological factors.

Methods

A retrospective cohort analysis was conducted on 97 patients with rectal adenocarcinoma who underwent surgical treatment at Erol Olçok Training and Research Hospital between January 2017 and December 2022. The inclusion criteria consisted of patients over 18 years of age and the absence of hematological disorders or concurrent inflammatory conditions. The patients' demographic data, tumor characteristics, surgical details, lymph node (LN) status, mtLNR, and survival outcomes were analyzed. The optimal cutoff value of mtLNR for predicting mortality was determined using receiver operating characteristic (ROC) curve analysis. Kaplan-Meier survival analysis was employed to estimate overall survival (OS) and disease-free survival (DFS), and differences between groups were evaluated using the log-rank test. The Cox proportional hazards model was used to calculate hazard ratios (HRs) for all-cause mortality. Statistical significance was set at p<0.05.

Results

The mean age of the patients was 70.31 ± 11.57 years, with 65.98% being male. Low anterior resection (LAR) was performed in 83.51% of the patients, and laparoscopic surgery was conducted in 26.8%. The median OS for the entire cohort was 24 months (range: 3-60). Patients were divided into two groups based on mtLNR, with the cutoff value set at 0.2183. A high mtLNR was significantly associated with poorer DFS and OS (p=0.021 and p=0.003, respectively). Moreover, patients with an mtLNR>0.2183 exhibited significantly higher rates of recurrence, lymphovascular invasion (LVI), and perineural invasion (PNI) compared to those with a lower mtLNR (all p<0.001). The optimal cutoff value of mtLNR predicted mortality with a specificity of 81.4% and a sensitivity of 48.1% (area under the curve (AUC) 0.662, p=0.012). Kaplan-Meier analysis showed a significant difference in survival between the two groups; the risk of all-cause mortality was 3.71 times higher in patients with mtLNR>0.2183 (p=0.002).

Conclusion

The mtLNR is a strong determinant of survival and prognosis in patients with rectal carcinoma. High mtLNR values are associated with worse survival outcomes and more aggressive tumor characteristics. The findings suggest that mtLNR should be considered in clinical decision-making processes. These results indicate that mtLNR could be a valuable prognostic tool in clinical decision-making.

## Introduction

Rectal cancer is a significant public health issue among malignant gastrointestinal tumors, affecting both developed and developing countries. As a subtype of colorectal cancer, which is the third most common cancer globally, rectal cancer is associated with high mortality rates. While surgical intervention is a fundamental approach in the treatment of rectal cancer, the presence of lymph node (LN) metastases is a critical factor influencing treatment outcomes. The spread of LN metastases directly impacts the cancer stage, disease progression, and patient survival [1-4].

Lymph node ratio (LNR), calculated as the ratio of the number of metastatic LNs to the total number of LNs removed, has increasingly been recognized as an important prognostic marker for solid tumors such as rectal cancer. LNR is recommended to enhance staging accuracy, especially in cases with an insufficient number of LNs removed. Although the traditional tumor, node, metastasis (TNM) staging system relies on the number of LN metastases, LNR offers a more comprehensive assessment by reflecting both the biological aggressiveness of the tumor and the effectiveness of the surgery [5-8]. 

Many studies have demonstrated that a high LNR correlates with reduced survival rates in rectal cancer patients. For example, an LNR greater than 10% has been linked to a significantly worse prognosis. Additionally, LNR is recognized as a reliable prognostic marker not only for rectal cancer but also for other solid tumors, including gastric, pancreatic, and breast cancers. Thus, LNR may help avoid stage shifts in cancer treatment and enhance decision-making in patient management [9-12]. Given that the count of LN metastases can be misleading and the number of removed LNs may vary by surgeon and surgical extent, highlighting the prognostic value of LNR is essential. In this context, a low LNR may reflect less aggressive tumor biology and potentially longer survival, while a high LNR indicates a higher metastatic burden and worse prognosis [13-15].

This study aims to assess the impact of LNR on survival and prognosis in rectal cancer patients. By evaluating if LNR is a more accurate prognostic marker than the number of LN metastases, we aim to improve staging and prognosis in rectal cancer treatment.

## Materials and methods

This study is a retrospective analysis conducted at the Department of General Surgery, Erol Olçok Training and Research Hospital, covering a total of 97 patients diagnosed with rectal adenocarcinoma between January 2017 and December 2022. The study population includes patients over 18 years of age. Inclusion criteria encompassed the absence of hematological disorders or conditions that could affect prognosis, non-use of medications that might influence hematological or biochemical parameters, and the absence of concurrent inflammatory or infectious pathologies. Patients with incomplete data, which could compromise the accuracy of survival analyses, were excluded from the study. Specifically, missing data related to critical variables such as survival times, tumor characteristics, and LN metrics were identified through a detailed review of medical records, and any cases lacking this essential information were systematically excluded to maintain the integrity of the analysis.

Ethical approval for the study was obtained from the Hitit University Faculty of Medicine Ethics Committee, with decision number 2023-179 dated December 26, 2023. Written and verbal consent was obtained from all participants, and the study adhered to the principles of the Helsinki Declaration.

In this retrospective study, various patient characteristics were meticulously documented from medical archive records. These characteristics included surgical type, demographic data (age and sex), disease-free survival (DFS), overall survival (OS), recurrence, mortality, tumor size (in cm), number of malignant LNs, total number of LNs, metastatic LNR (mtLNR), carcinoembryonic antigen (CEA), carbohydrate antigen 19-9 (CA19-9), TNM stage, lymphovascular invasion (LVI), and perineural invasion (PNI).

All statistical analyses were performed using IBM SPSS Statistics for Windows, version 26 (IBM Corp., Armonk, USA). Descriptive statistics for categorical variables were reported as counts and percentages, while numerical variables were presented as mean ± standard deviation or median (range) depending on the distribution pattern. The Shapiro-Wilk test was utilized to assess the normality of the data distribution.

To handle potential confounding factors, multivariate analysis techniques were employed. Correlations between variables were evaluated using Pearson correlation coefficients for normally distributed data and Spearman correlation coefficients for non-normally distributed data. Student’s t-test was used for comparing numerical variables with normal distribution (e.g., age), and the Mann-Whitney U test was applied for comparisons between groups for non-normally distributed variables (DFS, OS, tumor size, number of malignant LNs, total LNs, mtLNR, CEA, and CA19-9). Categorical variables such as sex, tumor location, and aortic calcification incidence were analyzed using the Chi-square test.

Missing data were managed by excluding cases with incomplete critical information, and the study accounted for potential biases during both study design and data analysis phases. The impact of confounders was controlled by including relevant variables in the multivariate models, and adjustments were made to mitigate their influence on the outcomes. Receiver operating characteristic (ROC) analysis was performed to determine the optimal mtLNR threshold for differentiating between survivors and deceased patients. Kaplan-Meier survival analysis was employed to predict the OS times of patient groups categorized by mtLNR levels. Statistical significance was established at p<0.05.

## Results

Out of the patients in the study, 81 (83.51%) underwent low anterior resection (LAR), while 26 (26.8%) had laparoscopic surgery. The average age of the participants was 70.31 ± 11.57 years, with 64 (65.98%) being male. The median OS for the cohort was 24 months, with a range of 3 to 60 months. Of the 120 patients initially considered, 23 were excluded due to missing data, lack of consent, or other reasons. The remaining 97 patients were classified into two groups: survivors (n=70) and non-survivors (n=27).

No significant differences were observed between the groups in terms of surgery type, use of laparoscopy, gender, tumor size, total number of LNs, CEA levels, CA19-9 levels, and TNM stages (p=0.782, p=0.253, p=0.571, p=0.961, p=0.246, p=0.260, p=0.512, and p=0.088, respectively) (Table 1). The average age of deceased patients was significantly higher than that of survivors (75.74 ± 10.97 years vs. 68.21 ± 11.18 years, p=0.004). The recurrence rate was 55.56% (15 patients) in the deceased group, compared to 17.14% (12 patients) in the survivors (p<0.001). Deceased patients had a median DFS of 10 months (range: 3-46), which was significantly shorter than that of survivors (p<0.001). Deceased patients had a significantly higher number of malignant LNs removed compared to survivors (p=0.006). The prevalence of LVI and PNI in deceased patients was 70.37% (19 patients) and 62.96% (17 patients), respectively, both of which were significantly higher than in the survivor group (p=0.029 and p=0.005, respectively).

**Table 1:** General characteristics of the participants and comparison between the alive and deceased groups APR: Abdominoperineal resection; LAR: Low anterior resection; LN: Lymph node; mtLNR: Metastatic lymph node ratio; OS: Overall survival; DFS: Disease-free survival; LVI: Lymphovascular invasion; PNI: Perineural invasion; CEA: Carcinoembryonic antigen; CA19-9: Carbohydrate antigen 19-9; TNM: Tumor, node, metastasis *p<0.05 indicates statistical significance

Variables		All Patients (n=97)	Alive (n=70)	Deceased (n=27)	Statistical Significance
Operation Type	APR	16 (16.49%)	12 (17.14%)	4 (14.81%)	0.782
	LAR	81 (83.51%)	58 (82.86%)	23 (85.19%)	
Laparoscopic	Open Approach	71 (73.20%)	49 (70.00%)	22 (81.48%)	0.253
	Laparoscopic	26 (26.80%)	21 (30.00%)	5 (18.52%)	
Age		70.31 ± 11.57	68.21 ± 11.18	75.74 ± 10.97	0.004*
Gender	Male	64 (65.98%)	45 (64.29%)	19 (70.37%)	0.571
	Female	33 (34.02%)	25 (35.71%)	8 (29.63%)	
DFS Duration (Months)		18 (3-60)	20 (11-60)	10 (3-46)	<0.001*
OS Duration (Months)		24 (3-60)	24 (12-60)	23 (3-53)	0.026*
Recurrence	No Recurrence	70 (72.16%)	58 (82.86%)	12 (44.44%)	<0.001*
	Recurrent Disease	27 (27.84%)	12 (17.14%)	15 (55.56%)	
Mortality	Alive	70 (72.16%)	-		-
	Deceased	27 (27.84%)			
Tumor Size (cm)		4.5 (0.2-12.5)	4.5 (0.2-12.5)	5 (2-8.1)	0.961
Malignant LNs		1 (0-13)	1 (0-11)	2 (0-13)	0.006*
Total LNs		12 (2-61)	11.5 (2-61)	13 (5-30)	0.246
mtLNR		0.08 (0-0.86)	0.05 (0-0.73)	0.20 (0-0.86)	0.011*
CEA		3.89 (0.5-2580)	3.9 (0.5-2580)	3.89 (1.28-592)	0.260
CA19-9		12.3 (0.2-192)	11.84 (0.3-192)	13.13 (0.2-86.8)	0.512
TNM	T1N0	4 (4.12%)	4 (5.71%)	0 (0.00%)	0.088
	T2N0	10 (10.31%)	10 (14.29%)	0 (0.00%)	
	T2N1	5 (5.15%)	5 (7.14%)	0 (0.00%)	
	T3N0	18 (18.56%)	12 (17.14%)	6 (22.22%)	
	T3N1	25 (25.77%)	16 (22.86%)	9 (33.33%)	
	T3N2	12 (12.37%)	8 (11.43%)	4 (14.81%)	
	T4N0	7 (7.22%)	6 (8.57%)	1 (3.70%)	
	T4N1	10 (10.31%)	7 (10.00%)	3 (11.11%)	
	T4N2	6 (6.19%)	2 (2.86%)	4 (14.81%)	
LVI	Negative	47 (48.45%)	38 (54.29%)	8 (29.63%)	0.029*
	Positive	50 (51.55%)	32 (45.71%)	19 (70.37%)	
PNI	Negative	58 (59.79%)	48 (68.57%)	10 (37.04%)	0.005*
	Positive	39 (40.21%)	22 (31.43%)	17 (62.96%)	

**Figure 1:**
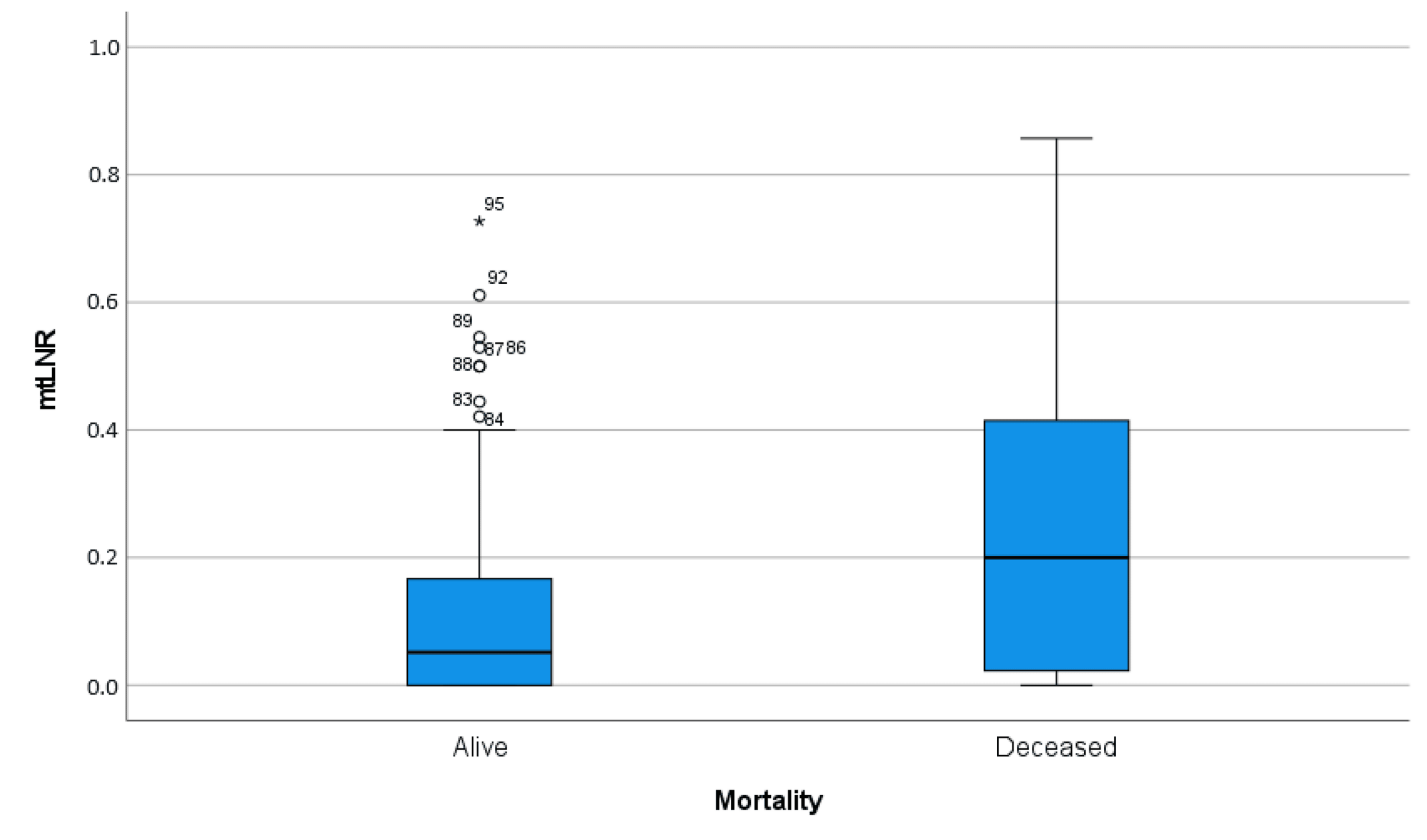
Boxplot diagram of mtLNR between alive and deceased groups mtLNR: Metastatic total lymph node ratio *: Extreme outlier

The median mtLNR was 0.05 (range: 0-0.73) in survivors, while it was 0.20 (range: 0-0.86) in deceased patients, and it was significantly higher in the deceased patients (p=0.011) (Figure 1).

**Figure 2:**
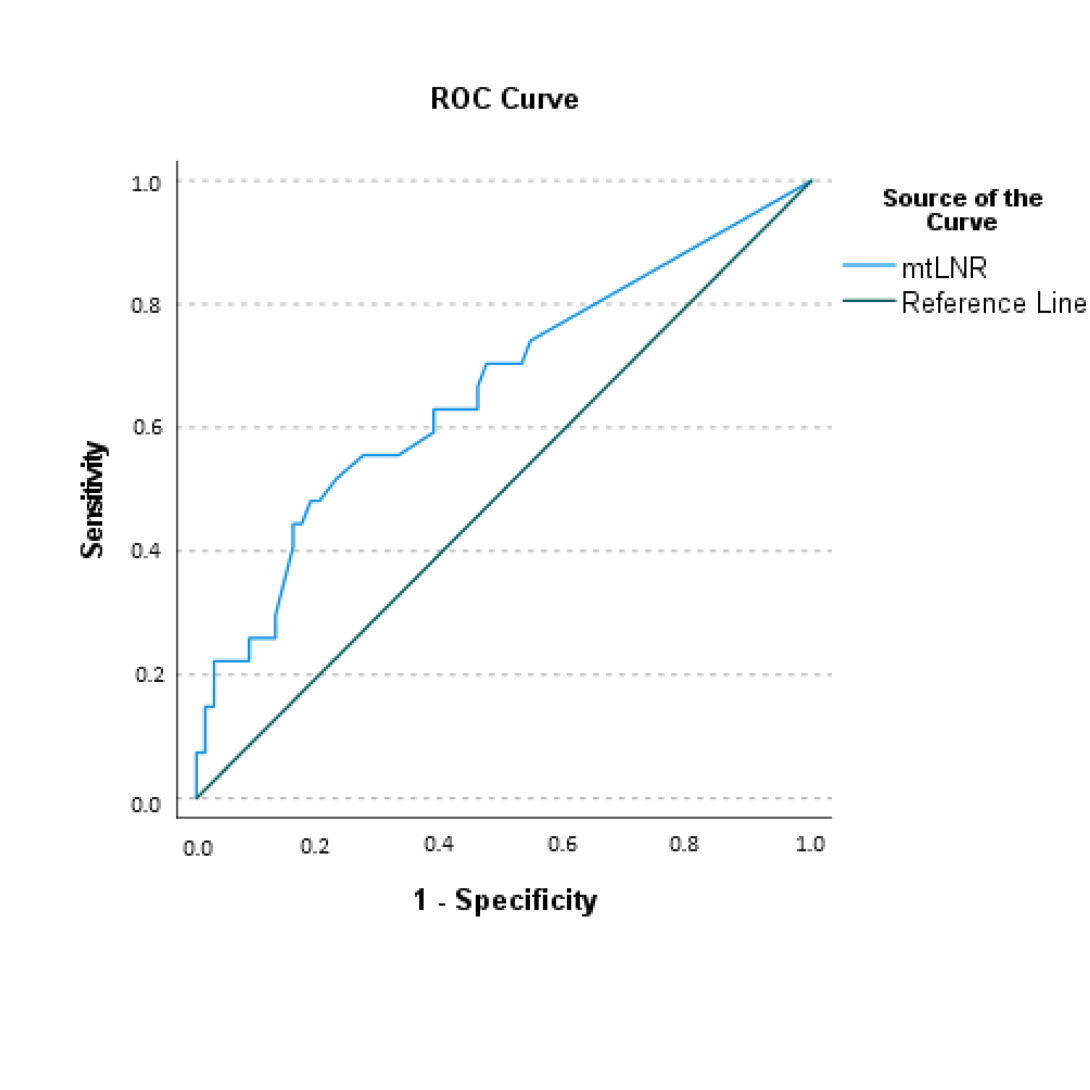
Receiver operating curve of mtLNR in the distinction of alive and deceased patients mtLNR: Metastatic lymph node ratio

Given the significant difference in mtLNR values between the two mortality groups, the optimal mtLNR cut-off value for distinguishing between survivors and deceased patients was evaluated using ROC analysis with area under the curve (AUC) and Youden index (AUC 0.662 (0.065), 95% CI 0.535-0.789, p=0.012). The optimal mtLNR value for predicting mortality was determined to be 0.2183, with 81.4% specificity, 48.1% sensitivity, 50.0% positive predictive value (PPV), 80.3% negative predictive value (NPV), and 72.2% test accuracy (OR 4.071, 95% CI 1.550-10.695, p=0.003) (Figure 2)(Table 2). An mtLNR exceeding 0.2183 was associated with a 3.71-fold increase in the risk of all-cause mortality (p=0.002).

**Table 2:** Diagnostic values of optimal mtLNR cut-off point mtLNR: Metastatic lymph node ratio; OS: Overall survival; DFS: Disease-free survival; PPV: Positive predictive value; NPV: Negative predictive value; SE: Standard error

Variables	Cut-Off	Diagnostic Values					ROC Analysis			Odds Ratio		
		Sensitivity	Specificity	PPV	NPV	Accuracy	Area (SE)	95% CI	p	OR	95% CI	p
mtLNR	≥0.2183	48.1%	81.4%	50.0%	80.3%	72.2%	0.662 (0.065)	0.535-0.789	0.012	4.071	1.550-10.695	0.003

When patients were divided into two groups according to the mtLNR cutoff, no significant differences were observed between the groups in terms of type of surgery, use of laparoscopy, age, gender, OS, tumor size, and CA19-9 level (p=0.219, p=0.308, p=0.335, p=0.683, p=0.323, p=0.635, and p=0.136, respectively). The mtLNR-positive group had shorter DFS, higher recurrence rates, and higher mortality rates (p=0.021, p<0.001, and p=0.003, respectively). Although the distribution of CEA differed between the groups, there was no difference in the distribution of CA19-9 (p=0.043 and p=0.136, respectively). In the mtLNR-positive group, the TNM stage was significantly more advanced, and the rates of LVI and PNI positivity were higher (p<0.001 for all) (Table 3).

**Table 3:** Patients characteristics and comparison results of groups according to mtLNR cut-off APR: Abdominoperineal resection; LAR: Low anterior resection; mtLNR: Metastatic lymph node ratio, OS: Overall survival; DFS: Disease-free survival; TNM: Tumor, node, metastasis; LVI: Lymphovascular invasion; PNI: Perineural invasion; CEA: Carcinoembryonic antigen; CA19-9: Carbohydrate antigen 19-9 *Significant difference between groups (p<0.05)

Variables		mtLNR<0.2183	mtLNR>0.2183	Statistical Significance
Operation Type	APR	10 (14.08%)	6 (23.08%)	0.291
	LAR	61 (85.92%)	20 (76.92%)	
Laparoscopic	Open Approach	50 (70.42%)	21 (80.77%)	0.308
	Laparoscopic	21 (29.58%)	5 (19.23%)	
Age		69.62±11.88	72.19±10.68	0.335
Gender	Male	46 (64.79%)	18 (69.23%)	0.683
	Female	25 (35.21%)	8 (30.77%)	
DFS Duration (Months)		19 (3-60)	13 (4-46)	0.021*
OSl Duration (Months)		27 (3-60)	19.5 (4-60)	0.323
Recurrence	No Recurrence	60 (84.51%)	10 (38.46%)	<0.001*
	Recurrent Disease	11 (15.49%)	16 (61.54%)	
Mortality	Alive	57 (80.28%)	13 (50.00%)	0.003*
	Deceased	14 (19.72%)	13 (50.00%)	
Tumor Size (cm)		4.5 (0.5-12.5)	4.5 (0.2-9)	0.635
CEA		3.4 (0.5-2580)	5.63 (0.92-592)	0.043*
CA19-9		11.49 (0.3-65)	15.95 (0.2-192)	0.136
TNM	T1N0	4 (5.63%)	0 (0.00%)	<0.001*
	T2N0	10 (14.08%)	0 (0.00%)	
	T2N1	4 (5.63%)	1 (3.85%)	
	T3N0	18 (25.35%)	0 (0.00%)	
	T3N1	18 (25.35%)	7 (26.92%)	
	T3N2	1 (1.41%)	11 (42.31%)	
	T4N0	7 (9.86%)	0 (0.00%)	
	T4N1	9 (12.68%)	1 (3.85%)	
	T4N2	0 (0.00%)	6 (23.08%)	
LVI	Negative	43 (60.56%)	3 (11.54%)	<0.001*
	Positive	28 (39.44%)	23 (88.46%)	
PNI	Negative	51 (71.83%)	7 (26.92%)	<0.001*
	Positive	20 (28.17%)	19 (73.08%)	

**Figure 3:**
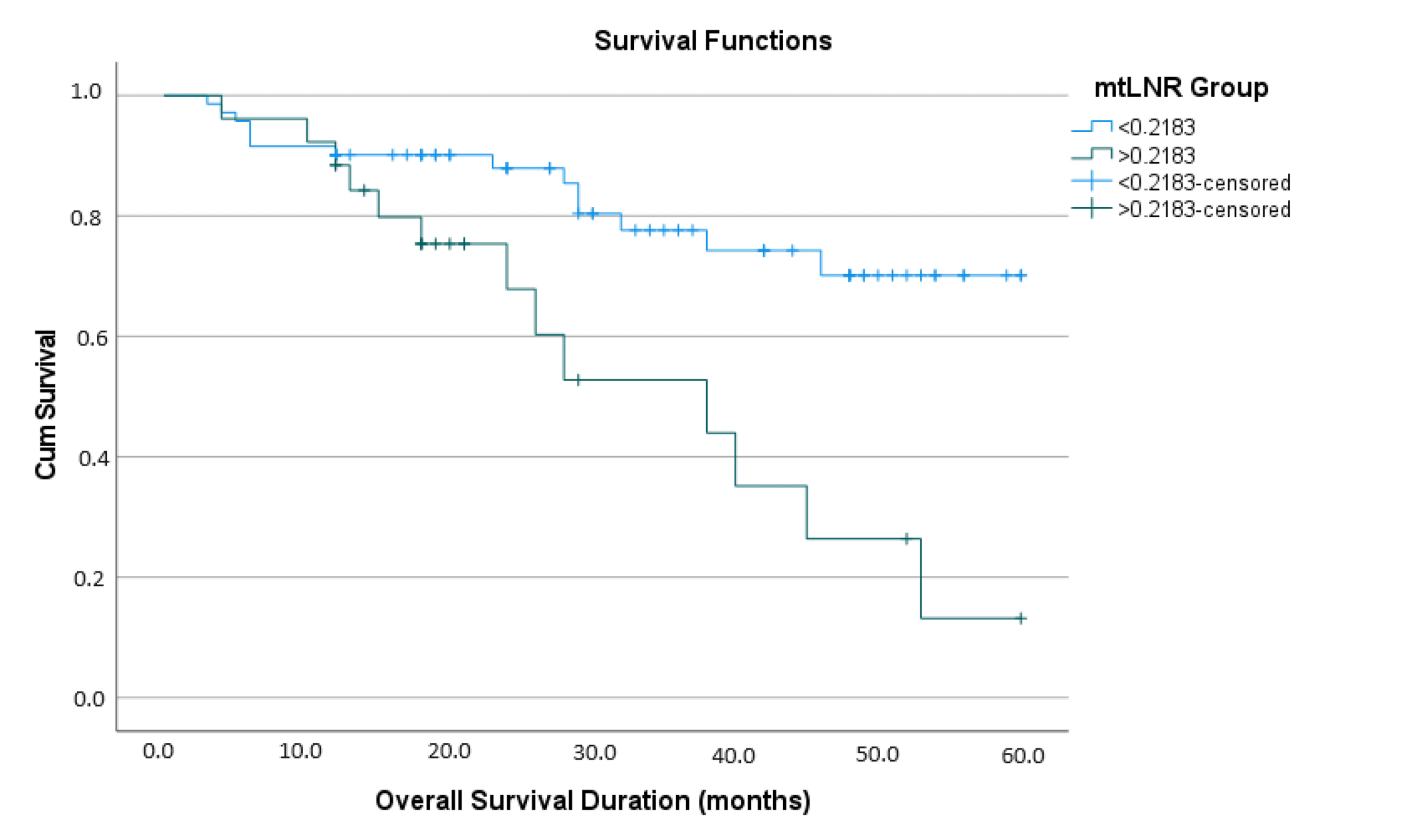
Kaplan-Meier analysis graph of groups according to mtLNR mtLNR: Metastatic lymph node ratio; OS: Overall survival

Kaplan-Meier survival analysis and the log-rank test indicated that the mean estimated OS expectancy for the whole cohort was 45.22 ± 2.29 months (95% CI 40.727-49.704). Patients with mtLNR<0.2183 had a mean estimated OS time of 49.404 ± 2.478 months (95% CI 44.547-54.262), while those with mtLNR>0.2183 had a mean estimated OS time of 34.614 ± 4.064 months (95% CI 26.650-42.579), a difference of approximately 15 months (p=0.001) (Figure 3).

At 60 months, the estimated survival rate was approximately 75% in the mtLNR-negative group, decreasing to less than 20% in the mtLNR-positive group (Table 4).

**Table 4:** Kaplan-Meier analysis estimated OS durations and log-rank test results mtLNR: Metastatic lymph node ratio; OS: Overall survival

mtLNR Group	Estimated Survival Duration	Std. Error	95% CI		Log Rank Test Significance
			Lower Bound	Upper Bound	
<0.2183	49.404	2.478	44.547	54.262	0.001
>0.2183	34.614	4.064	26.650	42.579	
Overall	45.215	2.290	40.727	49.704	

## Discussion

Our study highlights the significant association between high LNR and poorer survival outcomes, reinforcing the value of LNR as a prognostic marker in rectal cancer beyond traditional staging methods. However, it is important to consider the relatively low sensitivity of the mtLNR cutoff value reported in this study (48.1%), which may lead to a higher rate of false negatives. This limitation suggests that further research is needed to explore alternative cutoff values or the use of combination markers to enhance the prognostic accuracy of mtLNR in clinical practice. While the study provides valuable insights into the prognostic importance of LNR, its limitations, such as the single-center design and retrospective nature, should be considered when interpreting the results and applying them to broader clinical practice [2,6,7].

Our study observed that an mtLNR exceeding 0.2183 significantly increases the risk of mortality. This finding is supported by similar results in other studies. The literature emphasizes the prognostic value of LNR, especially in gastrointestinal cancers. For example, high LNR values have been shown to negatively impact survival and can be considered an independent prognostic factor beyond traditional TNM staging systems [3,4,9].

The extent and adequacy of lymph node dissection play a critical role in the success of rectal cancer treatment. Insufficient removal of lymph nodes can lead to incorrect staging of patients, which may negatively affect survival predictions. Our study demonstrated that patients with high mtLNR had more advanced TNM stages and higher rates of LVI and PNI. These findings reveal that high mtLNR is associated not only with the presence of lymph node metastases but also with the biological aggressiveness of the tumor [11,14].

Furthermore, mtLNR serves as a more accurate measure of both surgical intervention and tumor burden, going beyond just counting lymph node metastases. Particularly, in cases of extensive lymph node dissection, more accurate staging can be achieved, which may play a crucial role in determining treatment strategies. LNR assessment can provide consistent and comparable results even in cases where surgical practices vary across different centers [10,14,16].

Our study's findings demonstrate that LNR is a powerful determinant of survival beyond traditional TNM staging methods in rectal cancer patients. The literature indicates that high LNR values similarly negatively impact survival in other solid tumors (e.g., colon and gastric cancers). This suggests that LNR can be a significant prognostic marker not only for rectal cancer but also for various gastrointestinal cancers [5,11,17].

The results of our study indicate that high mtLNR has significant negative effects on both DFS and OS. Moreover, the high recurrence rates and the potential role of mtLNR in predicting these recurrences have been observed. Particularly, the presence of other clinical parameters indicating aggressive tumor biology (such as LVI and PNI) in patients with high LNR suggests the need for closer monitoring and tailored treatment strategies for these patients [7,9,17].

Finally, this study offers important implications for clinical practice regarding the prognostic value of mtLNR. LNR emerges as a superior marker compared to traditional methods based on the count of lymph node metastases. This finding underscores the need to consider LNR in the management and treatment planning of rectal cancer patients. Future research involving larger populations and multi-center studies may further reinforce the prognostic value of LNR [8,12,16].

Our study has some limitations. Firstly, it has a retrospective design, lacking some advantages of prospective studies. Since data were collected retrospectively from patient records, there is a possibility of missing or incorrect clinical information. Secondly, our study is single-center, which may limit the generalizability of the findings. Finally, our sample size is relatively small, which may reduce statistical power and require validation in a larger patient population.

In our study, a meticulous methodology was followed to minimize biases in patient selection, data collection, and analysis processes. To ensure the homogeneity of the patient population, we included patients over 18 years of age with a diagnosis of rectal adenocarcinoma, without hematological disorders, who were not on medications affecting hematological and biochemical parameters, and without concurrent inflammatory or infectious pathologies; patients with missing data were excluded. Standard protocols were adopted for data collection, and appropriate statistical methods were used to reduce the impact of missing data. Additionally, multiple statistical analyses were performed to control for potential confounding factors and enhance the accuracy of the results.

Our study's strengths include being one of the first to evaluate the impact of mtLNR on survival and prognosis in rectal cancer patients. Additionally, by determining the optimal cut-off value for mtLNR using ROC analysis, we provide a concrete threshold for clinical practice. Our study emphasizes the importance of accurately assessing the extent of lymph node dissection and metastatic burden.

## Conclusions

In conclusion, mtLNR is a strong determinant of survival and prognosis in rectal cancer patients. High mtLNR values are associated with poorer survival rates and more aggressive tumor biology. These findings suggest that mtLNR should be considered an important factor in clinical decision-making and patient prognosis prediction. Future research with larger sample sizes, multi-center, and prospective studies can help further elucidate the prognostic value of mtLNR.
